# Supplementary material for: Dynasore Protects Corneal Epithelial Cells Subjected to Hyperosmolar Stress in an In Vitro Model of Dry Eye Epitheliopathy
Source: Int J Mol Sci. 2023 Mar 1;24(5):4754. doi: 10.3390/ijms24054754 (PMC10003680; doi:10.3390/ijms24054754)
Supplement: Supplementary file 1 [file ijms-24-04754-s001.zip › ijms-2204047-supplementary.pdf]

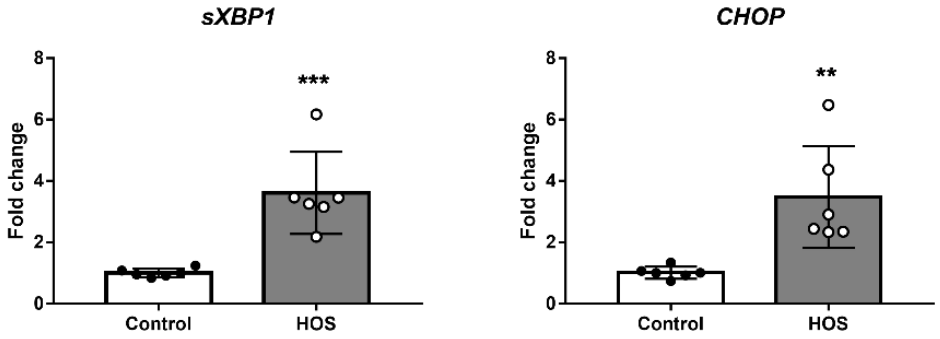

Figure S1. HOS induces activation of the unfolded protein response. Stratified HCLE cells were either incubated in normal DMEM or in hyperosmolar media by increasing DMEM NaCl concentration in 69 mM. After 24 h cells were processed for RNA isolation and qPCR analysis. Relative gene expression of *sXBP1* and *CHOP* was calculated with the $2^{-\Delta\Delta}$Ct method, using the levels of *ACTB* expression as housekeeping and the expression in control, untreated cells as the calibrator. The data are presented as mean ± standard deviation. **$p < 0.01$; ***$p < 0.0001$.
